# Isolated specialist or system integrated physician – different views on sickness certification among orthopaedic surgeons: an interview study

**DOI:** 10.1186/1472-6963-8-273

**Published:** 2008-12-23

**Authors:** Malin Swartling, Rolf Wahlström

**Affiliations:** 1 Department of Neurosciences, Rehabilitation Medicine, Uppsala University, Uppsala, Sweden; 2 Department of Public Health and Caring Sciences, Family Medicine and Clinical Epidemiology, Uppsala University, Uppsala, Sweden; 3 Department of Public Health Sciences, Division of International Health (IHCAR), Karolinska Institutet, Stockholm, Sweden

## Abstract

**Background:**

Sickness certification is a frequent and sometimes problematic task for orthopaedic surgeons.

Our aim was to explore how orthopaedic surgeons view their sick-listing commission and sick-listing practice.

**Methods:**

Semi-structured interviews with seventeen orthopaedic surgeons from five orthopaedic clinics in four Swedish counties. The focus was on the experiences of these physicians in relation to handling of sickness certification. Phenomenographic analysis was performed to reveal differences in existing views.

**Results:**

The orthopaedic surgeons' views on sick-listing seemed mainly to be a consequence of how they perceived their role in the healthcare system. Three categories were found: The "*isolated specialists"*, whose work and responsibilities were confined to the orthopaedic clinic, and did not really include sickness certification; the "*orthopaedic advisers"*, who saw themselves mainly as advice-givers in the general health care system and perceived sickness certification as part of their job; the "*system-integrated physicians"*, who perceived the orthopaedic clinic as one part of the healthcare system and whose ultimate goal was to get the patient well functioning in her life again with regained work ability, seeing sick-listing as one of the instruments to achieve this. Some informants described difficulties in handling conflicting opinions with patients in relation to the need for sick-leave.

**Conclusion:**

Orthopaedic surgeons certify a large proportion of total sickness benefits. Some orthopaedic surgeons may certify sickness benefits sub-optimally for patients and society due to a narrow view of their role in the health care system or due to poor skills in handling discordant opinions with the patient. This problem can be addressed at the level of the individual physician and at the system level.

## Background

In Sweden, the number of people on sick leave more than doubled from 1997 to 2003, and such absence is still high in 2008 even if it is gradually decreasing [1]. In order for a person to receive sickness benefits a licensed physician needs to certify him or her sick as a basis for an insurance officer to take the formal decision. Sickness certification is a frequent task among orthopaedic surgeons [2]. More than 80% have consultations including sick-listing at least six times per week, which make them the most exposed of all physicians in Sweden [2]. Many physicians find sick-listing problematic [2,3] and 53% of orthopaedic surgeons, experience problems regarding sick-listing at least 1–5 times a week, and 15% experience conflicts with patients regarding sickness certification equally often. A larger proportion of orthopaedic surgeons than other types of physicians find it problematic to manage the double role of being the patient's doctor and a medical expert for authorities [3].

General Practitioners (GPs) also sick-list frequently [2,3], but they have been shown to differ from orthopaedic surgeons by sick-listing longer [4], and sick-listing via the telephone to a larger extent [5] as well as differ among themselves [3] with regard to their sick-listing practices. Among 19 GPs in a study based on the same interview guide as in this current study, qualitatively different views regarding sickness certification were found [6], but how orthopaedic surgeons perceive their sick-listing commission, sick-listing practices and their role in sickness certification has to our knowledge not been studied.

Our aim was to identify and describe the views among Swedish orthopaedic surgeons on sick-listing practice and on the sick-listing commission, and to discuss this in comparison with GP's views.

## Methods

### Data collection

Five orthopaedic clinics in four Swedish counties with a total of 108 specialists (98 males) were targeted. An updated list of physicians was obtained from the head of each clinic. After checking age and specialist status in the national list of licensed physicians, 32 orthopaedic surgeons (comprising about 30% of the specialists at each clinic for a fair distribution) were approached by post.). The aim was to recruit approximately 20 interviewees who varied regarding age, sex, site of hospital, sub speciality and research experience in order to maximize the potential difference in views among interviewees. Reminders were handled by e-mail and telephone. Three substitute recruitments were done. Thirty-five orthopaedic surgeons were asked to participate and 20 agreed. However, two could not be interviewed due to time constraints, and one interview recording had to be excluded due to extremely poor sound quality. Participants and those not wanting to participate did not differ in any important respect. The studied seventeen orthopaedic surgeons (15 males) were 44–66 years old (median 46). Eight of them worked at one of the two university hospitals included, and nine were employed at referral hospitals.

Seventeen individual interviews were performed in June-August 2004 by the first author only in order to avoid inter-interviewer reliability (also called dependability) problems [7]. Fifteen of the interviews were conducted at the workplaces of the participants, and two were performed over the telephone. A semi-structured interview guide with open-ended questions was used (Additional file 1). The guide focused on the physician's own experience of handling cases involving sickness certification, and descriptions of specific examples. The interview guide had previously been used in a similar study of GPs [6]. If needed, probing questions were used to help the interviewee elaborate and reflect. The interviews lasted 21–60 minutes (median 35 minutes), and they were audio recorded after verbal consent and transcribed verbatim. The transcripts were verified against the tapes, NVivo 2.0 software was used for data management. The identity of the physicians was known only to the interviewer.

### Data analysis

The analysis was performed using a phenomenographic approach [8]. This qualitative research approach was initially developed in Swedish pedagogic research [9], but has increasingly been used also in health care research [6,8,10-12]. In the phenomenographic approach, it is proposed that a phenomenon is understood in a limited number of qualitatively different ways [13] and that normally 15–20 informants capture the existing variation in views and experiences in a homogenous group of people [14,15].

All transcripts were carefully read several times by both authors to obtain a general overview of the contents. Thereafter, the first author (MS) selected the most significant statements made by each informant regarding a certain domain (area of interest within the phenomenon studied) that had caught our attention, in order to create a short but representative version of the entire content concerning that particular domain. The authors then independently compared these condensed expressions under each domain to find similarities and differences that could justify grouping into different categories of descriptions i.e., different ways of perceiving or understanding specific phenomena. A category of description will in the following synonymously also be called a "view". Throughout the analysis, we applied an iterative process that involved switching back and forth between the whole transcript and the condensed versions. No predetermined categories were used. We subsequently compared our individual categorisations and found that the level of agreement was high. We had found the same categories and there was only slight disagreement on two or three of the transcripts, and those differences were resolved through discussions and with reference to the total material in the transcripts [15]. It has been suggested that working towards informed consensus in this manner is a way towards assuring accuracy of the analysis [15]. The procedure was repeated for each domain.

Three domains were studied: (i) the role of the orthopaedic surgeon in the health care system; (ii) handling of consultations involving conflicting opinions of the physician and the patient about the need for sickness certification; (iii) the sick-listing commission (the assigned task, or the remit of issuing sickness certificates). The third domain was found and defined in our previous study of GPs views on sick-listing [6] and found again in this material; the second domain was also found there but presented in a less explicit way, while the first domain emerged during the analysis. A few quotations from relevant parts of the interviews are presented in the results section to illustrate different categories. It should be noted that in most cases such excerpts cannot include more than one or two of the aspects of a category description.

The outcome of a phenomenographic study also includes the structural relationships of the categories of descriptions – the outcome space – which often show a hierarchical order [11,16,17]. Some views (categories of descriptions) are composed of few aspects, compared to views higher in the hierarchy, where several aspects are included. We have used the term 'inclusive views' for more complex categories of description and have presented our results in order of such inclusiveness.

The study was approved by the Ethics Committee of Uppsala University.

## Results

The interview guide was structured to explore the informants views on their sick-listing commission and their view on good sick-listing practices. However, we found that it was the surgeons' perceptions of their own role in the health care system that seemed to have a more extensive effect on certifying sickness absence, and, therefore we chose to analyse this emergent domain. This was in contrast to our study on GPs [6], where their role in the health care system was not a issue and thus did not seem to affect the GPs' views of their role in sickness certification. The domain "sick-listing commission" and "handling disagreements with patients on sickness certification" were found among the GPs, as well as among the orthopaedic surgeons, and the same categories of description were found in the two groups.

### 1. Role in the system

We found three different ways in which the orthopaedic surgeons perceived their role in the health care system in relation to sickness certification, and we call those categories *the isolated specialists*, *the orthopaedic advisers*, and *the system-integrated physicians*.

#### 1a. The isolated specialists

The work of an orthopaedic surgeon involves assessing patients for surgery, performing the actual operations, and conducting follow-ups. The focus of the orthopaedic work is within the orthopaedic clinic, which is not perceived as part of a broader health care system, and thus these doctors make little effort to communicate with social security staff or primary care providers. Sick-listing is not perceived to be a task for orthopaedic surgeons, but as something they are forced to handle against their will, and want to get rid of. Everything that is not directly linked to surgery could just as well be done by someone else.

Sick certificates should in principle be issued only on orthopaedic indications. However, rather than discussing with a patient who wants to be on sick leave contrary to the professional judgment of the physician, the doctor allows the patient's opinion regarding work ability to steer the decision about certification. This happens because the surgeon either wants to be of service or finds it too unpleasant and time consuming to argue with the patient. Sickness certification is perceived as conflict filled and emotionally taxing.

I did sick-list her for quite some time, but finally I said [...] if you want to get yourself an early retirement, you'll have to go to your GP. I was nice to her for a while and gave her sick notes [even though there was no orthopaedic reason]. (Dr B)

I think it's rather unpleasant [issuing sick notes], and [...] it's difficult and it's sort of our own [physicians'] fault that the level of sickness absence is high. (Dr E)

#### 1b. The orthopaedic advisers

The orthopaedic clinic is perceived to be neither isolated from, nor part of the rest of the health care system. The patients come to the orthopaedic clinic on a visit from the general system to get advice and help with orthopaedic complaints. Certification of sick leave is mentioned in quite technical terms and is regarded as part of the job for orthopaedic surgeons. Moreover, it is not perceived as a particularly conflict-laden task, although some of the surgeons in this category want to get rid of sick-listing because of the extra work it entails.

She started to cry and said "I can't work when I'm like this." And I said, "Well, I guess you'll have to, because your body is fully functional, and having just a little pain can't make you completely unfit for work." And yeah, she wasn't satisfied; she had come on a referral note from a doctor. So I said that she would just have to go back to that doctor and discuss it with him. (Dr J)

#### 1c. The system-integrated physicians

The orthopaedic clinic is an integrate part of the health care system. Facilitating the transfer of a patient from the orthopaedic clinic to the next station in the care system is perceived to be part of the orthopaedic surgeon's job. It is always important to consider the long-term implications of an injury or operation and to communicate these to the patient and the local social insurance office at an early stage. When the orthopaedic surgeon feels the orthopaedic clinic has fulfilled its task, the patient should be transferred to primary care. The ultimate goal is to help the patient to become well functioning in her own life again with regained ability to work, and this can be promoted by proper management of the sickness certification instrument.

But we usually just sort of pilot them through this injury period, and then I refer them to primary care. All [patients], where there is a problematic situation, that one really has to get into, to get them going again. And then I don't extend their sick leave, but they get it from their GP. (Dr S)

### 2. Handling conflicting opinions

We found three different views concerning how the orthopaedic surgeons handle conflicting opinions about the need for sickness certification, and we call these *directed by the patient, compromising*, and *directed by professional judgement*:

#### 2a. Directed by the patient

Disagreements about the need for sick leave create an internal conflict for orthopaedic surgeons, who do not see how they can question patients without wasting time and emotional energy, and therefore they let the wishes of the patients determine whether or not to issue a sick note. The orthopaedic surgeons who hold this view find sickness certification very difficult and unpleasant, and hence they want to get rid of it. All of the respondents we call *isolated specialists *held this view, as did some of the system-integrated physicians.

*It [not issuing a sick note] costs too much time and energy. Mainly time I think – no both – kind of mental energy. (Dr E, isolated specialist)*.

A lot of times you end up in conflict, not with the patient, but with your own ideas [...] It depends a lot on what the patients themselves think, and that's frustrating when you're dealing with sickness certification. [...] it depends mostly on what the patients themselves think, what they think they can manage [...] You can't get patients back [to work] earlier than they want to themselves. It's almost impossible. (Dr D, system-integrated physician)

#### 2b. Compromising

The orthopaedic surgeons try to compromise when their opinions differ from those of their patients regarding the need for sick leave. This view was expressed by all of the surgeons in the *orthopaedic adviser group *and also by a few of those in the system-integrated category.

Sometimes you have to compromise so you won't lose the patient's trust completely. So it has to be a dialogue [with the patient], right, to get the patient to go back to work. (Dr. O, orthopaedic adviser)

#### 2c. Directed by professional judgement

When a patient and an orthopaedic surgeon judge the need for sick leave differently, a sick note should not be issued in contradiction to the professional judgement of the orthopaedic surgeon. The reason for that is that the responsibility of the physician does not end with a good outcome of surgery, but instead extends to the long-term well-being of the patient. We found this view only among the physicians identified as having a "*system integrated*" view of their role in the health care system. If an agreement cannot be reached with the patient, the physician has to act in an authoritative way.

*He [a car mechanic] had a healed forearm fracture with no signs at all of reduced function. All he said was that he was in pain so he needed to be on sick leave. I told him I didn't think it was good for him or any 24-year-old to stay at home and just hang around, that he really ought to go back to work or find another job. Then he got really mad and rushed out angry, and was rude. But I mean, (sigh), I've kind of learned to just shrug my shoulders, and after about four minutes I don't think about it any longer. If you do that, most patients pull themselves together and go to work and get a normal life. (Dr A, system-integrated physician*).

### 3. The sickness certification commission

We use the word "commission" here to mean a task that is entrusted to someone. Three different views on the sick-listing commission were identified;

#### 3a) the patient's commission

which focuses on the interests of the patient and is clearly less concerned with the interests of society;

#### 3b) the society's commission

which focuses on the interests of society and its responsibility to rehabilitate patients back to work;

#### 3c) the integrated commission

which combines the interests of the patient and society.

When I issue a sick note? It's on instructions from the patient. (Dr E, patient's commission)

And I have to try to follow the instructions of the social insurance offices and applicable rules the best I can. [...] it's only reasonable that we believe the patients, believe in what they say. We should always do that, but we have to evaluate [their situation] in relation to, um, laws and rules and our own professional knowledge. [...] (Dr C, society's commission)

*But in the cases when I say no [to issuing a sick note], it's kind of like I feel it's my obligation towards society and the system, to safeguard it's human values. It shouldn't have to be misused. So, so either I protect the patient from society or society from the patient. (Dr G, integrated commission*).

## Discussion

We found the orthopaedic surgeons' views on sick-listing to be, to a large extent, a consequence of their view of their role in the health care system. We found a hierarchy of three such views, with the least inclusive view being, "the isolated specialist", who does not want to deal with sick-listing at all, and the most inclusive view being "the system integrated physician", where sick-listing is seen in the context of an integrated health system. Views on how to handle discordant opinions with the patient on the need for sickness certification ranged from "patient directed" to "directed by professional judgement".

The views on the orthopaedic surgeons role in the system, resemble how some anaesthesiologists understand their work as investigated by Larsson in an interview study in Swedish hospitals [12]. "The isolated specialist", is similar to Larsson's "professional artists", who show a fairly narrow perception of anaesthesiology. Also, the "system integrated physician", who includes serving the patient in a system comprising more than the orthopaedic clinic, is similar to Larsson's "servant" anaesthesiologists who serve the entire hospital in order to serve the patient. Physicians with more inclusive views presumably have more options [11,12] to handle sick-listing situations, and this may be beneficial for both the doctors and their patients. This is similar to what Sandberg [14] described in another group of professionals, where engineers with a broader understanding of their work were judged by their colleagues to be more competent on the job. For the orthopaedic surgeons, a transition to more inclusive views would mean adopting a broader way of understanding [11,12,15] their role in the health care system including more aspects of the patient as a person, as well as sickness certification as a therapeutic instrument.

For the sick-listing practice of orthopaedic surgeons, it seems that not only is the way they perceive their role in the health care system of importance, but also their view of how to handle situations in which they disagree with the patient about the need for sick leave. Some avoid open conflicts with patients, which makes them "directed by the patient", i.e. rather than by their own professional judgement.

We believe that the views we refer to as "compromising" and "directed by professional judgement" are better for patients, physicians, and society than is the case with the "directed by the patient" view, where the physicians' issue sick notes against their professional judgment, and – as it often seems – also against the rules and regulations of the health care system. But obviously to *know *if one view is better than another, when it comes to patient outcome, other studies including quantitative methods are needed. Orthopaedic surgeons, who hold the least inclusive views on their role in the health care system ("isolated specialist") and are "directed by the patient" in the case of discordant opinions, may not function as gatekeepers in relation to sickness benefits, as is intended by policy makers in the sickness insurance system, nor optimally help patient's rehabilitation back to work.

Previous research has shown that individual physicians can adopt more inclusive views after an educational intervention [10,18]. For such a change to occur, the existing conceptions of the learner must be challenged [8], which calls for interactive educational strategies [19,20]. Simultaneous change of view *and *change of practice have been shown after an educational intervention [18], but it could not be shown that the practice changed *as a consequence *of the changed view. System change can also be considered. e.g. by limiting the time an orthopaedic surgeon may sick-list and letting for example a GP or, as suggested by Scottish GPs, a special insurance physician, take over after a set period of time [21].

By using the phenomenographic approach we aimed at getting a deeper understanding of the physicians' views of phenomena related to sick-listing, beyond stated attitudes. It has been shown repeatedly that attitudes (stated opinions) show ambiguous relations to behaviour [22,23]. Similar to Dall'Alba and Sandberg, we assume that views or the way one understands or perceives the phenomenon have a closer relationship to practice behaviour [11,24] than attitudes do.

The trustworthiness of findings should be illuminated in qualitative studies [25]. By asking the orthopaedic surgeons to describe, in their own words, how they manage real cases involving sickness certification, we received material that was close to their actual practice and where their way of presentation to a great extent reflected their views. In addition, we believe that our structured analysis of the interview material has given the results a reasonable degree of credibility, which is further supported by the dual categorisation done by two researchers and the negotiated consensus [26]. The transferability of our findings to other contexts in Sweden could be assumed to be reasonably high as the situation for sick-listing orthopaedic surgeons is similar all over the country and as we interviewed doctors in different types of settings. However, caution should be observed when attempting to extrapolate our results to other countries. Notwithstanding, the consistency of our findings can be questioned, because the practices of physicians might be influenced by changes in the social environment and in the regulations for issuing sick notes. However, we believe that the categories of description in our study have been interpreted at a level that is not directly affected by such external changes. Quantification of different views among orthopaedic surgeons and the extent to which differences in views are reflected in outcomes of patient care would be useful.

Some of the interviews were as short as half an hour, but despite the short time there was enough material for the analysis. Informants not sharing their views so easily were interviewed for a longer time.

Participants were easily recruited, 35 letters yielded 20 volunteers. Those not wanting to participate did not differ in any important respect, why we judge participation bias to be low.

The interview guide was piloted on both GPs [6] and on orthopaedic surgeons, revised and tested again on GPs, but not on orthopaedic surgeons, which, in hindsight, might have been a good idea. The guide was probably better adapted to exploring the GPs' perceptions on sick-listing. However, using the same guide to study both categories of doctors facilitated comparability of the results, and has clearly brought out the differences in their views of sick-listing, and how the orthopaedic surgeons perceive their role in the health care system. In the study of GPs their own role in the health care system was not even mentioned [6]. In the eyes of the interviewed GPs, sickness certification was obviously part of their job. They didn't even reflect on it. For orthopaedic surgeons sickness certification was not perceived by all as part of their job, why they all spent time explaining what really was the work of an orthopaedic surgeon as a background to how they viewed sickness certification. The work of a GP and an orthopaedic surgeon differs a lot and this could be the reason for their different ways of talking about sickness certification.

The interview guide included queries about things making good sick-listings more difficult. It would have been helpful if it had also considered matters that make that task *easier*. The views found on the sick listing commission (patient's, society's and integrated) have previously been found among Swedish GPs. However, contrary to the GPs, it seems that for the orthopaedic surgeons there is no correlation between their views on the sick-listing commission and their perceptions of the other domains studied [6].

Other researchers have provided clear evidence that the training of communication skills has a positive impact on the ability of medical students and physicians to handle difficult situations [27,28], although we have not found such studies on handling potential sick-listing cases. Communication skills training may help physicians to handle situations of discordant opinions on the need for sickness certification, which is often perceived as difficult. To design and evaluate such training is a suggestion for future research.

## Conclusion

Orthopaedic surgeons prescribe a large proportion of total sickness benefits. From the perspectives of both society and individual patients, it is possible that some orthopaedic surgeons may prescribe sickness benefits sub-optimally due to a narrow view on their role in the health care system, or due to insufficient skills in handling disagreements with patients. This issue can be addressed at the levels of both the physician and the health care system.

## Competing interests

The authors declare that they have no competing interests.

## Authors' contributions

MS coordinated the study, conducted all the interviews, verified the transcripts against the tapes, and drafted the manuscript. MS and RW read all the transcripts and performed the analysis. RW commented on the manuscript. Both authors conceived and designed the study, and read and approved the final manuscript.

## Pre-publication history

The pre-publication history for this paper can be accessed here:



## Supplementary Material

Additional file 1 **Appendix**. Click here for file
