# Supplementary material for: Isolated specialist or system integrated physician – different views on sickness certification among orthopaedic surgeons: an interview study
Source: BMC Health Serv Res. 2008 Dec 23;8:273. doi: 10.1186/1472-6963-8-273 (PMC2651137; doi:10.1186/1472-6963-8-273)
Supplement: Additional file 1 — Appendix. [file 1472-6963-8-273-S1.doc]

#### Appendix 1. The interview guide used in the semi-structured interviews

| **Opening wordings for exploration of views:** |
| --- |
| GENERAL  What comes to your mind when you hear the word “sickness certification”? |
| PRACTICE  Please tell me about a recent consultation where sickness certification was considered  - a case where you feel comfortable with the way you handled it  - a case where you feel less comfortable with the way you handled it |
| THE TASK OF SICKNESS CERTIFICATION  What are your feelings about the task of sick-listing? |
| THE IDEAL  When you sick-list a patient, do you ever feel you would have preferred to do something different from what you did and in that case; why and how? |
| BARRIERS  Is there anything else that makes it difficult for you to sick-list people the way you would prefer to – and in that case, what? |
| CONCLUDING GENERAL  Is there anything else you would like to add about sickness certification or any aspect of sickness certification? |

A complete interview guide with probing questions can be obtained via the first author.
